# In France, distance from hospital and health care structure impact on outcome after arthroplasty of the hip for proximal fractures of the femur

**DOI:** 10.1186/s13018-023-03893-4

**Published:** 2023-06-09

**Authors:** Mathieu Levaillant, Louis Rony, Jean-François Hamel-Broza, Julien Soula, Benoît Vallet, Antoine Lamer

**Affiliations:** 1 Univ. Lille, CHU Lille, ULR 2694 - METRICS : Évaluation des technologies de santé et des pratiques médicales, 59000 Lille, France; 2 Centre Hospitalier Universitaire d’Angers, Angers, France; 3 University Angers, Angers, France

**Keywords:** Volume–outcome relationship, Hip arthroplasty, Hospital, Quality indicator, Surgery, Data reuse

## Abstract

**Background:**

Hip arthroplasty is a frequently performed procedure in orthopedic surgery, carried out in almost all health structures for two main issues: fracture and coxarthrosis. Even if volume–outcome relationship appeared associated in many surgeries recently, data provided are not sufficient to set surgical thresholds neither than closing down low-volumes centers.

**Question:**

With this study, we wanted to identify surgical, health care-related and territorial factors influencing patient’ mortality and readmission after a HA for a femoral fracture in 2018 in France.

**Patients and methods:**

Data were anonymously collected from French nationwide administrative databases. All patients who underwent a hip arthroplasty for a femoral fracture through 2018 were included. Patient outcome was 90-day mortality and 90-day readmission rate after surgery.

**Results:**

Of the 36,252 patients that underwent a HA for fracture in France in 2018, 0.7% died within 90-day year and 1.2% were readmitted. Male and Charlson comorbidity index were associated with a higher 90-day mortality and readmission rate in multivariate analysis. High volume was associated with a lower mortality rate. Neither time of travel nor distance upon health facility were associated with mortality nor with readmission rate in the analysis.

**Conclusion:**

Even if volume appears to be associated with lower mortality rate even for longer distance and time of travel, the persistence of exogenous factors not documented in the French databases suggests that regionalization of hip arthroplasty should be organized with caution.

**Clinical relevance:**

As volume–outcome relationship must be interpreted with caution, policy makers should not regionalize such surgery without further investigation.

**Supplementary Information:**

The online version contains supplementary material available at 10.1186/s13018-023-03893-4.

## Introduction

Hip arthroplasty (HA) is one of the most frequently performed procedures in orthopedic surgery [1]. Evolution of engineering, expertise of the surgical procedure by orthopedic surgeons and the relatively long follow-up of this surgery have made prosthetic hip surgery very safe [2].

HA is performed in two nosological settings: elective surgery for coxarthrosis or acute surgery mainly for femoral neck fracture. Although the procedure are the same, the populations involved are different, with more comorbidities in the case of traumatology [3, 4].

For this common pathology, hip arthroplasty is performed in all health care structures offering orthopedic and traumatological surgery. Population characteristics may vary according to the health care structures and geographic localization. There is a lack of data concerning the outcomes after HA [5]. Even if volume–outcome relationship appeared positive in many surgeries in the last decades [6–9], data provided are not sufficient to set thresholds for surgical activity, nor to close low-volume centers, with a dramatic impact on inequalities in access to care [10]. A recent meta-analysis focusing on HA highlighted inconsistent effect of volume on patient outcome [11] illustrating the need of further investigations.

The objective of this study was to identify surgical, health care-related or territorial factors influencing patient’ mortality and readmission after a HA for a femoral fracture in 2018 in France.

## Methods

This study was conducted using the French national health data system (SNDS), including health insurance claim SNIIRAM [Système national d’information inter-régimes de l’assurance maladie] and hospital discharge databases PMSI [Programme de médicalisation des systèmes d’information] [12]. The SNIIRAM [13, 14] anonymously collects demographic data (i.e., sex, age, place of residence, vital status) and all data concerning reimbursed care. Such data do not contain any information about clinical results. The PMSI covers all admissions, including inpatient care and day-hospital care in every public or private hospital in France. Hospital stay is documented with individual-level data about the date of admission, length of stay, hospital code number and outcome (i.e., discharge, hospital transfer, death). Principal diagnosis, defined as the main reason for admission, and associated diagnoses, related to comorbidities, are collected and coded according to the French version of the International Statistical Classification of Diseases and Related Health Problems, 10th Revision [ICD-10]). Medical and surgical procedures are documented with the *Classification Commune des Actes Médicaux (CCAM)*, a French terminology.

A unique national identification number for each patient allows the linkage of all admissions for the same patient, which provides detailed information on health consumption for more than 98% of the population living in France.

### Study population

All patients cared for in a hospital between the January 1, 2018 and the December 31, 2018 for a hip arthroplasty were included. Both total and hemiarthroplasty were identified with the CCAM codes NEKA010 to NEKA021 reported in the Additional file 1: Table S1 and according to the International Classification of Disease 10th edition code S72 (i.e., femur fracture). Patients’ with a bilateral arthroplasty performed on the same day were excluded. Only the first surgery was considered for analysis for patients who had two hip arthroplasties within the inclusion period.

### Primary and secondary outcomes

The primary outcome was all-cause mortality during the 90 days after surgery. Secondary outcome was 90-day all-causes readmission following the arthroplasty hospital stay. When multiple readmissions were identified, only the first readmission was considered.

### Defining variables

Hospitalization-related variables collected were age, sex, length of stay, duration between admission and surgery, Charlson comorbidity index (CCI) [15] and the public or private status of the health facility (public, private nonprofit, private commercial, territorial collectivity). Charlson comorbidity index was calculated using Quan et al. [16] updated version for ICD-10 administrative data. Charlson was calculated based on the comorbidities coded within the arthroplasty procedure’s initial hospital stay.

Time of travel and distance between patients’ latest known residence and hospital was obtained by matching patients’ and hospital postal code with an C++ API provided by project OSRM, based on OpenStreetMap data [17, 18]. Time of travel was expressed as a qualitative value, above and lower 30 min.

### Statistical analysis

Quantitative variables were expressed as mean and standard deviation. Qualitative variables were expressed as number and percentage.

Analysis was performed using Cox survival models, including several covariates. Age, sex, CCI, surgical indication, time between admission and surgery, length of stay, hospital procedural volume, hospital juridic status, distance and time of travel between patient city of residency and health care facility were included as fixed-effect covariates. The hospital was included as a random effect covariate for taking into account the potential specificities of each care center independently from the volume effect. In these Cox models, age was categorized in four different subgroups (corresponding to quartiles): <73 y.o., 73–82 y.o., 83–89 y.o., > 89 y.o. Facility activity volume was calculated from the sum of all total and hemi-hip arthroplasties performed in 2018 for a femoral proximal fracture and then expressed in quartiles for analyses. Results are presented as Hazard Ratio with their 95% confidence intervals.

All *p* values presented were for a 2-sided test, and the threshold of significance was defined as a *p* value < 0.05. These statistical analyses were performed using SAS Enterprise Guide 9.4 software (SAS Institute Inc., Cary, NC, United States).

## Results

Between January 1, 2018 and December 31, 2018, 36,252 patients underwent an acute surgery for a hip arthroplasty after a femur fracture in French hospitals. Among them, 73.3% were female, with a mean age of 81.9 y.o. (± 10.5) and with a mean length of stay of 9.9 days (± 6.7). 245 died in the 90-day following the surgery (0.7%) and 468 during the 1-year post-surgery (1.3%). Most surgeries were performed in public health facilities (73.2%), followed by commercial (20.6%) then nonprofit ones (5.4%). The average time of travel from home to hospital was 26.4 min (± 57.5), and the mean distance of 28.2 km (± 86.2).

The demographic characteristics of the patients are reported in Table 1.

**Table 1:** Description of the population

	*N* =	36,252
Age	81.9	10.5
Female	26,564	73.3%
Delay between admission and surgery	2.3	2.7
Length of stay	9.9	6.7
Charlson comorbidity index		
0	22,988	63.4%
1	9433	26.0%
2	2914	8.0%
3+	917	2.5%
Hospital juridic status		
Public facility	26523	73.2%
Territorial collectivity	318	0.9%
Private nonprofit	1940	5.4%
Private commercial	7471	20.6%
Distance	28.2	86.2
Time of travel	26.4	57.5
Procedural volume	156.9	266.4
Deceased		
Within 30 days	120	0.3%
Within 90 days	245	0.7%
Within 1 year	468	1.3%
Readmitted in a hospital facility		
Within 30 days	434	1.2%
Within 90 days	968	2.7%

Qualitative data are presented with number and percentage; quantitative as mean and standard deviation

### Relationship between 90-day mortality, patient’ and hospital characteristics

Patients who died within 90 days were significantly older (85.4 vs. 81.9, *p* < 0.001), with higher Charlson comorbidity index (1.00 vs. 0.50, *p* < 0.001). They also lived closer to the facility where they were operated (15.0 vs. 26.5 min, *p* < 0.001), surgery that took place in smaller surgical units (mean procedural volume 87.7 vs. 157.4, *p* < 0.001).

In the multivariate analysis, the factors associated with a higher 90-day mortality were age (either for 73–82 y.o., HR 2.48; 83–89 y.o., HR 3.90, and above 89 y.o. HR 4.96; *p* < 0.01) and Charlson comorbidity index score (p < 0.001). Female gender (HR 0.57, *p* < 0.001) along with a higher volume facility (third quartile HR 0.37, *p* = 0.036 and forth quartile HR 0.09, *p* < 0.001) and a shorter length of stay (HR 0.97, *p* = 0.033) appeared protective for 90-day mortality. Delay between admission and surgery, health facility juridic status, time of travel and distance was not found significantly associated with 90-day mortality in the multivariate analysis. Analysis also showed a significant hospital effect (*p* < 0.001), independently of all other covariates considered (especially independently from the hospital characteristics such as the hospital volume) (Table 2).

**Table 2:** Cox regression of factors associated with 90-day post-hip arthroplasty all-causes mortality

	Hazard ratio	IC95%		*p* value
Female	0.57	0.44	0.73	< 0.001
Age				< 0.001
Age < 76	Ref			
Age 77–84	2.48	1.63	3.78	< 0.001
Age 85–89	3.90	2.59	5.86	< 0.001
Age > 89	4.96	3.29	7.49	< 0.001
Charlson comorbidity index				< 0.001
Charlson 0	Ref			
Charlson 1	1.90	1.42	2.54	< 0.001
Charlson 2	3.91	2.78	5.49	< 0.001
Charlson 3 and above	4.42	2.58	7.57	< 0.001
Health facility juridic status				0.535
Public facility	Ref			
Territorial collectivity facility	0.00	0.00		0.982
Private nonprofit	1.28	0.44	3.74	0.650
Private commercial	0.60	0.27	1.32	0.203
Procedural activity volume				0.001
First quartile—[1;54[	Ref			
Second quartile—[55;89[	0.62	0.29	1.34	0.221
Third quartile—[90;145[	0.37	0.15	0.94	0.036
Forth quartile—[146;1435[	0.09	0.02	0.38	0.001
Delay between admission and surgery	1.04	0.99	1.10	0.142
Length of stay	0.97	0.95	1.00	0.033
Time of travel above 30 min	0.57	0.32	1.02	0.057
Distance	1.00	0.99	1.01	0.406
Hospital random effect	158.52			< 0.001

### Relationship between 90-day all-causes readmission, patients’ and hospital characteristics

Within the 30 days after surgery, 1.2% of patients having undergone a hip arthroplasty were readmitted in a hospital facility, and 2.6% after 90 days. The primary reason for readmission was mechanical complications of internal joint prostheses [ICD-10 T84] (*n* = 118, 10.4% after 90 days), followed by other medical care, mostly related to oncological or palliative issues [ICD-10 Z51 and Z49] (*n* = 129, 11,4%), arthritis [ICD-10 M00] (*n* = 30, 2.7%), heart failure [ICD-10 I50] (2.7%) and fracture of femur [ICD-10 S72] (*n* = 28, 2,5%).

Patients readmitted within 90 days were significantly younger (80.5 y.o. vs. 82.0 y.o., *p* = 0.010) but they had a significantly higher Charlson comorbidity index (0.67 vs. 0.50, *p* < 0.001). Patients readmitted in a health facility within 90-day live closer to it than those who were not (18.3 min vs. 26.7 min, *p* < 0.001), and were initially operated in smaller facilities (procedural volume 86.9 vs. 159.1, *p* < 0.001).

In the multivariate analysis, the factors associated with a higher 90-day readmission rate were mainly Charlson comorbidity index (CCI 1 HR 1.3, CCI 2 HR 1.4, CCI 3 and above HR 1.9; *p* < 0.001). Female gender (HR 0.82, *p* < 0.001) and shorter length of stay (HR 0.98, *p* < 0.001) were associated with less 90-day all-causes readmission. Patients’ age, health facility juridic status and activity volume, time of travel and distance were not associated with a different 90-day readmission rate. Analysis did not show a significant hospital effect (*p* = 0.057) (Table 3).

**Table 3:** Cox regression of factors associated with 90-day all-causes readmission

	Hazard ratio	IC95%		*p* value
Female	0.82	0.72	0.93	0.002
Age				0.069
Age < 76	Ref			
Age 77–84	1.15	0.98	1.34	0.092
Age 85–89	1.24	1.05	1.47	0.010
Age > 89	1.09	0.91	1.31	0.340
Charlson comorbidity index				< 0.001
Charlson 0	Ref			
Charlson 1	1.25	1.10	1.43	0.001
Charlson 2	1.37	1.12	1.67	0.002
Charlson 3 and above	1.89	1.35	2.63	0.000
Health facility juridic status				0.479
Public facility	Ref			
Territorial collectivity facility	0.03	0.00	1.99E+128	0.982
Private nonprofit	0.79	0.60	1.04	0.089
Private commercial	0.96	0.72	1.27	0.772
Procedural activity volume				0.069
First quartile—[1;54[	Ref			
Second quartile—[55;89[	0.90	0.72	1.13	0.379
Third quartile—[90;145[	0.77	0.59	0.99	0.044
Forth quartile—[146;1435[	0.93	0.66	1.33	0.702
Delay between admission and surgery	0.98	0.95	1.01	0.270
Length of stay	0.98	0.97	1.00	0.006
Time of travel above 30 min	1.06	0.85	1.31	0.613
Distance	1.00	1.00	1.00	0.548
Hospital random effect	16.30			0.057

## Discussion

The objective of this study was to describe the outcome of 36,252 patients who underwent hip arthroplasty following femoral neck fracture in 2018 in France, as well as the influence of travel time between home and hospital, and hospital characteristics on their outcome.

Male and Charlson comorbidity index appeared associated with either a higher 90-day all-causes mortality and readmission. On the opposite, a shorter length of stay appeared associated with both a lower 90-day all-causes mortality and readmission. Age was associated with a higher 90-day mortality, while high-volume facilities was associated with a lower 90-day mortality. No association were found between such variables and 90-day readmission.

Our results are similar with the one published by Maceroli et al. [19], who found that high volume was associated with mortality but not with revision rates, as did Mahoney [20].

A positive volume–outcome relationship was identified toward 90-day mortality, independently of the patients characteristics, with a protective effect in the multivariate analysis for the third and fourth activity volume structures, congruently with previous studies [21]. It may be noticed that in our study, academic and non-academic facilities were not separated even if a possible effect was previously published [22]. Another bias of the volume classification used is the assessment of only a year volume and not of the upward or downward trend in surgical activity, while Mukhtar et al. showed a higher effect of the trend than of the instant volume [23].

Time of travel above 30 min and distance between residency place and health facility were not associated neither with mortality nor with readmission rate in the multivariate analysis. Our results are not congruent with previous work. As to our knowledge such an analysis was not performed only on acute hip arthroplasty, we can only compare our results with literature based on other surgeries. A few studies showed that travel distance effect is mainly a reflect of travel to higher centers, and that high-volume center may overwhelm travel distance effect [24]. However, such distance and time of travel effect have mostly been identified for highly technical surgeries such as oncology [25–27]. Indeed, easy access to an orthopedic surgery service anywhere in France may explain the lack of effect of travel time on mortality and readmission, as only a few patients have long time travel, in particular in emergency situation such as acute surgeries [28].

We highlighted an effect of volume, but also a hospital random effect, suggesting that neither patients’ characteristics nor volume nor juridical status can explain on their own the difference in patients mortality among each facility investigated. A few variables, sometimes identified as relevant in the literature and not included in our model may explain this effect: surgeons’ activity volume [11], surgeons’ age [29] or even the geographic distribution of facilities.

Finally, socio-demographic characteristics of the patients and their territory may influence their outcome, as demonstrated by Gonzalez et al. [30] on aneurysm surgery, study where ethnicity and socio-economic status interacted with the surgical volume of each facility.

Our study is the first of its kind, exploring the whole French population undergoing a total or hemi-arthroplasties arthroplasty after a proximal femur fracture. However, our study should be considered with limitations. In particular, the fact that we analyzed all arthroplasties without differentiating between intermediate arthroplasties and arthroplasties for femoral neck fractures is a limitation of our results. However, since this is a global analysis, it is likely that the proportion of intermediate and total arthroplasties is smoothed out across all centers. Moreover, intermediate arthroplasties are often reserved for “fragile” patients, whereas total arthroplasties are often reserved for patients in “good general condition” that should be included in our analysis using the Charlson comorbidity index. Furthermore, even if a center varied from the norm by offering only one type of arthroplasty for the same indication, the volume of data would smooth out the effect of that center. Another limitation stand with data not available in the French database used, in particular the use of cement or non-cemented prostheses, even if literature suggests few differences in post-surgery outcome, such as cement impact.

A final limitation lies in the content of the database itself. The data are only available at the level of the institutions and not of the surgeons themselves, even though the literature has shown that the effect of volume can also be linked to the practitioner himself [31]. However, the use of the hospital level is supported by studies on the logic of “failure to rescue” or on the effect of the entire care team on the improvement of patient outcome [32].

Our results should enable public decision makers to take a step back from the current French and global dynamics of excessive centralization of surgical activities [33–35]. The existence of a significant random effect in all the models studied suggests the existence of an unidentified input in our analyses that impacts patient outcome. The impact on mortality and readmission cannot therefore be studied simply on the basis of patient characteristics and the volume of activity of the hospital. An unexplained feature between hospital and outcome may explain why among many countries that has experimented centralization of surgical activity, all did not succeeded. Two reasons are described in the scientific literature about centralization: the first is that low-volume and high-volume centers do not gather the same populations. Gani et al. [10] showed in 2017 that ethnic minorities, elderly patients and patients with many comorbidities may have more difficulty accessing high-volume centers, which increases inequalities in access to care. Secondly, even if a volume–outcome relationship was identified in most of surgical fields and countries, studies that evaluated the effect of centralization and threshold showed that having only high-volume centers had adverse effects and might not improve patient outcome. For example, Stitzenberg et al. [36] reported that a marked increase in traveling distance observed after the centralization of pancreatic surgery posed a significant obstacle to accessing quality care and increased inequalities in care access for specific populations—mainly in rural states [37].

## Conclusion

Surgical volume is associated with a better outcome after a hip arthroplasty within 90 days and 1 year, with no effect demonstrated on hospital readmission. Such results are consistent even after adjustment for distance and time of travel, but the persistence of exogenous factors not documented in the French databases suggests that regionalization of hip arthroplasty should be organized with caution. Quantity should not be opposed with quality. Such facility reorganization should be investigated with more precisions.

## Supplementary Information

**Additional file 1.** Table S1.

## Data Availability

The data that support the findings of this study are available on request from the corresponding author, ML.
