# Supplementary material for: In France, distance from hospital and health care structure impact on outcome after arthroplasty of the hip for proximal fractures of the femur
Source: J Orthop Surg Res. 2023 Jun 9;18:418. doi: 10.1186/s13018-023-03893-4 (PMC10257255; doi:10.1186/s13018-023-03893-4)
Supplement: Supplementary file 1 — Additional file 1. Table S1. [file 13018_2023_3893_MOESM1_ESM.docx]

**In France, distance from hospital and health care structure impact on outcome after arthroplasty of the hip for proximal fractures of the femur**

***Additional file 1: Table S1 - Description of the CCAM codes used to include patients in the study***

| **NEKA010** | Remplacement de l'articulation coxofémorale par prothèse totale, avec renfort métallique acétabulaire et reconstruction fémorale par greffe | Replacement of the coxofemoral joint by total prosthesis, with metallic acetabular reinforcement and femoral reconstruction by graft |
| --- | --- | --- |
| **NEKA011** | Remplacement de l'articulation coxofémorale par prothèse fémorale cervicocéphalique et cupule mobile | Replacement of the coxofemoral joint by cervicocephalic femoral prosthesis and mobile cup |
| **NEKA012** | Remplacement de l'articulation coxofémorale par prothèse totale, avec reconstruction acétabulaire ou fémorale par greffe | Replacement of the coxofemoral joint by total prosthesis, with acetabular or femoral reconstruction by graft |
| **NEKA013** | Remplacement de l'articulation coxofémorale par prothèse totale après arthrodèse coxofémorale | Replacement of the coxofemoral joint by total prosthesis after coxofemoral arthrodesis |
| **NEKA014** | Remplacement de l'articulation coxofémorale par prothèse totale, avec renfort métallique acétabulaire | Replacement of the coxofemoral joint by total prosthesis, with metallic acetabular reinforcement |
| **NEKA015** | Remplacement de l'articulation coxofémorale par prothèse totale après ostéosynthèse, ostéotomie ou prothèse cervicocéphalique du fémur | Replacement of the coxofemoral joint by total prosthesis after osteosynthesis, osteotomy or cervicocephalic prosthesis of the femur |
| **NEKA016** | Remplacement de l'articulation coxofémorale par prothèse totale, avec ostéotomie de la diaphyse du fémur | Replacement of the coxofemoral joint by total prosthesis, with osteotomy of the diaphysis of the femur |
| **NEKA017** | Remplacement de l'articulation coxofémorale par prothèse totale, avec abaissement de la tête du fémur dans le paléoacétabulum [paléocotyle] | Replacement of the coxofemoral joint by total prosthesis, with lowering of the head of the femur in the paleoacetabulum [paléocotyle] |
| **NEKA018** | Remplacement de l'articulation coxofémorale par prothèse fémorale cervicocéphalique | Replacement of the coxofemoral joint by cervicocephalic femoral prosthesis |
| **NEKA019** | Remplacement de l'articulation coxofémorale par prothèse totale après arthrodèse coxofémorale, avec renfort métallique acétabulaire | Replacement of the coxofemoral joint by total prosthesis after coxofemoral arthrodesis, with acetabular metal reinforcement |
| **NEKA020** | Remplacement de l'articulation coxofémorale par prothèse totale | Replacement of the coxofemoral joint by total prosthesis |
| **NEKA021** | Remplacement de l'articulation coxofémorale par prothèse totale, avec abaissement de la tête du fémur dans le paléoacétabulum [paléocotyle] et ostéotomie de réaxation ou d'alignement du fémur | Replacement of the coxofemoral joint by total prosthesis, with lowering of the head of the femur in the paleoacetabulum [palaeocotyle] and osteotomy of reaxation or alignment of the femur |
